# Mitochondrial Dysfunction and **β**-Cell Failure in Type 2 Diabetes Mellitus

**DOI:** 10.1155/2012/703538

**Published:** 2011-11-09

**Authors:** Zhongmin Alex Ma, Zhengshan Zhao, John Turk

**Affiliations:** ^1^Division of Experimental Diabetes and Aging, Department of Geriatrics and Palliative Medicine, Mount Sinai School of Medicine, New York, NY 10029, USA; ^2^Division of Endocrinology, Metabolism and Lipid Research, Department of Medicine, Washington University School of Medicine, St. Louis, MO 63110, USA

## Abstract

Type 2 diabetes mellitus (T2DM) is the most common human endocrine disease and is characterized by peripheral insulin resistance and pancreatic islet **β**-cell failure. Accumulating evidence indicates that mitochondrial dysfunction is a central contributor to **β**-cell failure in the evolution of T2DM. As reviewed elsewhere, reactive oxygen species (ROS) produced by **β**-cell mitochondria as a result of metabolic stress activate several stress-response pathways. This paper focuses on mechanisms whereby ROS affect mitochondrial structure and function and lead to **β**-cell failure. ROS activate UCP2, which results in proton leak across the mitochondrial inner membrane, and this leads to reduced **β**-cell ATP synthesis and content, which is a critical parameter in regulating glucose-stimulated insulin secretion. In addition, ROS oxidize polyunsaturated fatty acids in mitochondrial cardiolipin and other phospholipids, and this impairs membrane integrity and leads to cytochrome *c* release into cytosol and apoptosis. Group VIA phospholipase A_2_ (iPLA_2_ **β**) appears to be a component of a mechanism for repairing mitochondrial phospholipids that contain oxidized fatty acid substituents, and genetic or acquired iPLA_2_ **β**-deficiency increases **β**-cell mitochondrial susceptibility to injury from ROS and predisposes to developing T2DM. Interventions that attenuate ROS effects on **β**-cell mitochondrial phospholipids might prevent or retard development of T2DM.

## 1. Introduction

Type 2 diabetes mellitus (T2DM) is the most common human endocrine disease and is reaching pandemic proportions [1]. Predisposition to T2DM is affected both by genetic and acquired factors, and there are contributions from many genes and environmental influences that are incompletely understood [1, 2]. It is becoming clear that the progressive failure of pancreatic islet **β**-cells is a central component of the development and progression of T2DM [3]. Normally, pancreatic islet **β**-cells respond to increased metabolic demands by increasing their mass and insulin synthetic and secretory activity, as demonstrated both in rodent models of obesity without diabetes and in nondiabetic obese humans.

Most humans who are obese do not develop diabetes, and T2DM develops only in those who are unable to sustain compensatory **β**-cell responses to increasing metabolic stress [4]. The United Kingdom Prospective Diabetes Study (UKPDS) has clearly demonstrated that the progressive nature of T2DM reflects an ongoing decline in **β**-cell function without a change in insulin sensitivity [5]. Longitudinal studies of subjects who eventually develop T2DM reveal a progressive rise in serum insulin levels in the prediabetic phase that is followed by a decline in serum insulin levels upon development of fasting hyperglycemia [1]. Many T2DM patients ultimately require therapy with exogenous insulin in the later stages of the disease because endogenous insulin production becomes insufficient to maintain acceptable levels of glycemia despite ongoing therapy with other antidiabetic agents, including sulfonylureas and metformin, *inter alia* [3]. 

Reductions in both **β**-cell mass and function contribute to the pathogenesis of **β**-cell failure in human T2DM [6, 7]. Several studies have demonstrated that glucose-stimulated insulin secretion is lower in islets from T2DM patients compared to control islets [8, 9]. In addition, islets from T2DM subjects exhibit both structural and functional abnormalities and fail to reverse hyperglycemia when transplanted into diabetic mice under conditions in which equivalent numbers of control human islets do so [9]. Interestingly, T2DM human islets secrete significantly higher amounts of insulin in response to arginine and glibenclamide than in response to D-glucose, suggesting that T2DM **β**-cell insulin secretory defects reflect a relatively selective loss of responsivity to glucose compared to other insulin secretagogues [10]. 

Moreover, it has been demonstrated that the ATP content of islets from T2DM subjects fails to increase normally upon acute stimulation with glucose. Consequently, their ATP/ADP ratio rises to values only about 60% of that in control islets, and this is likely to account for or contribute to the blunted or absent glucose-stimulated insulin secretory responses of T2DM islets [11]. Mitochondria in T2DM **β**-cells exhibit both morphologic and functional abnormalities that are not observed in control **β**-cells [11]. Together, these findings indicate that human T2DM **β**-cells exhibit abnormalities in glucose metabolism and in mitochondrial structure and function that result in impaired ATP production and glucose-stimulated insulin secretion [7]. 

Accumulating evidence indicates that progressive reduction in **β**-cell mass also contributes to the overall decline in **β**-cell functional capacity in the pathogenesis of T2DM. Early observations indicated that **β**-cell volume is significantly reduced in T2DM islets [12–14]. More recent studies with postmortem and surgical specimens of human pancreata have characterized changes in **β**-cell mass that occur during the evolution of T2DM [6, 15]. One such study based on specimens from 124 autopsies revealed a 63% lower **β**-cell volume in obese T2DM subjects compared to nondiabetic, weight-matched control subjects and a 41% lower **β**-cell volume in lean T2DM subjects compared to nondiabetic lean control subjects. Another study revealed a 40% lower **β**-cell mass in subjects with elevated fasting blood glucose levels compared to weight-matched control subjects with fasting euglycemia, which suggests that reductions in **β**-cell mass may not be confined to late-stage T2DM but may rather occur progressively throughout the prediabetic phase and continue after the onset of impaired glucose tolerance and then hyperglycemia [6]. Moreover, the decreased **β**-cell volume observed in subjects with fasting hyperglycemia is associated with increased **β**-cell death by apoptosis [6]. Evidence also indicates that the loss of **β**-cells is selective among islet cell types in the evolution of T2DM and that comparable losses of islet *α*-cells do not occur [15]. Together, these findings demonstrate that progressive structural and functional abnormalities occur in islets during the development of T2DM. 

The mechanisms that underlie the progressive development of **β**-cell failure during the evolution of T2DM are not fully understood at present [3]. Identifying the factors involved and characterizing the mechanisms by which they lead to **β**-cell failure would be important steps in elucidating the pathogenesis of T2DM and identifying potential targets for therapeutic interventions designed to retard or prevent these processes. Both genetic and acquired factors contribute to **β**-cell failure in T2DM [16], and, among the acquired factors, glucotoxicity, lipotoxicity, altered islet amyloid polypeptide (IAPP) processing, advanced glycation end-products (AGEs), and increased inflammatory cytokines have been suggested to contribute to **β**-cell injury [1, 7, 17–20]. 

Although many mechanisms are proposed to underlie effects of these factors, a unifying theme is that production of reactive oxygen species (ROS) induced by metabolic stress represents a common pathway of injury in the cascade of events that ultimately results in **β**-cell failure [3, 21–27]. Activation of a series of stress-response pathways by ROS has been reviewed elsewhere [28–30]. The purpose of our paper is to provide a brief overview of how mitochondrial ROS affect mitochondrial membrane phospholipids, including cardiolipin, and how this might lead to **β**-cell mitochondrial failure and ultimately result in T2DM. Recent advances in complex lipid analyses by mass spectrometry permit detailed molecular characterization of the effects of pathophysiologic states on mitochondrial cardiolipin species [31–34], and this provides a powerful tool with which to increase our understanding of these processes and to identify potential targets for therapeutic intervention.

## 2. Mitochondria Are the Most Important Cellular Source of ROS in ***β***-Cells

Oxidative stress can arise from various sources [35], and ROS appear to be produced in larger amounts by islets from T2DM patients than by those from nondiabetic subjects [23, 36–38]. Accumulating evidence indicates that obesity and hyperglycemia are associated with increased ROS production [22, 39]. Although ROS are generated in peroxisomes, for example, by cytochrome P450- and NADPH oxidase-catalyzed reactions, and in other nonmitochondrial loci, the major source of ROS production in cells is the mitochondrion [40].

**Figure 1:**
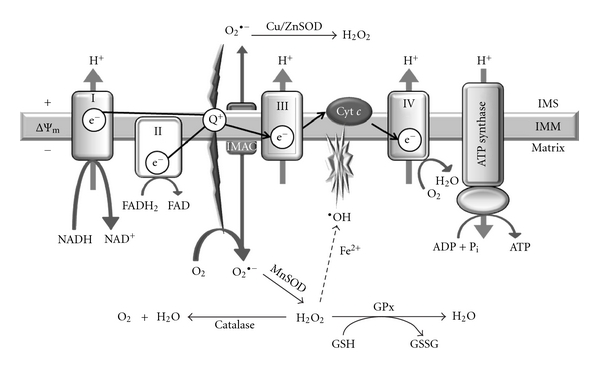
Mitochondrial ROS production and defense. The electron transport chain consists of four protein complexes (I–IV) and the ATP synthase located in the inner mitochondrial membrane (IMM). The activity of complex I converts NADH to NAD^+^, and the activity of complex II converts succinate to fumarate. Complexes I, III, and IV transport protons (H^+^) across the membrane, and complexes I and III generate superoxide anion radical (  O_2_^•−^) during the electron transfer process. O_2_^•−^ can naturally dismutate to hydrogen peroxide (H_2_O_2_) or is enzymatically dismutated by matrix manganese superoxide dismutase (MnSOD). O_2_^•−^ is not membrane permeable but can pass through inner membrane ion channel (IMAC) and is dismutated to H_2_O_2_ by Cu/ZnSOD in the intermembrane space (IMS)/cytoplasm. H_2_O_2_ is detoxified in the matrix by catalase and the glutathione peroxidase (GPx). Alternately, H_2_O_2_ can react with metal ions to generate via Fenton chemistry (dash line) the highly reactive hydroxyl radical (•OH) that can initiate the peroxidation of the inner mitochondrial membrane phospholipids, such as cardiolipin. Cyt. *c*: cytochrome *c*; IMS: intermembrane space; GSH: glutathione; GSSG: glutathione disulfide; ΔΨ_*m*_: membrane potential.

Electron flow through the mitochondrial electron-transport chain is carried out by four inner membrane-associated enzyme complexes (I–IV), cytochrome *c,* and the mobile carrier coenzyme Q. Molecular species of ROS include superoxide anion (O_2_^•−^), hydrogen peroxide (H_2_O_2_), and the hydroxyl radical (•HO), *inter alia*. The electron-transport chain continually generates small amounts of superoxide anion radicals, principally through complexes I and III [41]. Superoxide production increases substantially in the settings of obesity and hyperglycemia [22, 39]. Superoxide radicals are normally removed by Mn^2+^-superoxide dismutase (MnSOD), which dismutates O_2_^•−^ to produce H_2_O_2_ that is then reduced to water by catalase or glutathione peroxidase (GPx) at the expense of glutathione. When rates of H_2_O_2_ generation exceed those of its removal, H_2_O_2_ accumulation can result in production of the highly reactive hydroxyl radical in the presence of Fe^2+^ via the Fenton reaction and via the Haber-Weiss reaction of O_2_^•−^ and •HO (Figure 1). 

 If they are not rapidly eliminated, ROS can injure mitochondria by promoting DNA fragmentation, protein crosslinking, and peroxidation of membrane phospholipids and by activating a series of stress pathways [29]. Indeed, **β**-cell mitochondria in islets from T2DM subjects have been found to exhibit morphologic abnormalities that include hypertrophy, a rounded rather than elliptical shape, and higher density compared to **β**-cell mitochondria in islets from control subjects [11, 42]

## 3. ROS Trigger Apoptosis via Oxidation of Mitochondrial Inner Membrane Phospholipids in ***β***-Cells

The onset of T2DM is accompanied by a progressive decrease in **β**-cell mass that results from a marked increase in **β**-cell apoptosis [6, 7, 43], and mitochondria are known to play a pivotal role in regulating apoptotic cell death [44]. Proapoptotic stimuli induce release of cytochrome *c* from mitochondria into the cytoplasm, where cytochrome *c* participates in apoptosome formation that results in caspase-9 activation and subsequent activation of the executioner caspases 3, 6, and 7 that dismantle the cell during apoptosis [44].

Cytochrome *c* release from mitochondria is a key step in the initiation of apoptosis [45] and appears to result from direct action of ROS on the mitochondrial phospholipid cardiolipin [46, 47]. Cardiolipin is a structurally unique dimeric phospholipid exclusively localized in the inner mitochondrial membrane (IMM) in mammalian cells and is essential for maintaining mitochondrial architecture and membrane potential and for providing support to proteins involved in mitochondrial bioenergetics [48, 49]. Cytochrome *c* is anchored to the outer surface of the inner mitochondrial membrane by electrostatic and hydrophobic interactions with cardiolipin [50]. During the early phase of apoptosis, mitochondrial ROS production is stimulated, and cardiolipin is oxidized. This destabilizes the interaction with cytochrome *c*, which then detaches from the membrane and is released into the cytoplasm through pores in the outer membrane [46, 50]. 

Cardiolipin is particularly susceptible to oxidation because it is enriched in polyunsaturated fatty acid (PUFA) residues, especially linoleate (C18:2), which contain a bisallylic methylene group from which hydrogen is easily abstracted to provide a center for formation of a hydroperoxy radical via interaction with molecular oxygen. Linoleic acid (C18:2) is the most abundant fatty acid substituent of cardiolipin in most mammalian tissues [51], and rat pancreatic islet cardiolipin, for example, contains 89.5% PUFA and 71% linoleate [52]. Mitochondrial cardiolipin is also a target of the proapoptotic protein tBid, which is a Bcl-2-family member produced from Bid by the activation of caspase-8. This results in activation of the mitochondrial death pathway upon induction of apoptosis via engagement of death receptors [53]. Cardiolipin serves as a mitochondrial target of tBid, which promotes pore formation in the outer mitochondrial membrane by Bax or Bak in a process that is inhibited by Bcl-2 or Bcl-XL [54]. 

The mitochondrial phospholipid cardiolipin is thus a central participant in regulating apoptosis triggered by both the mitochondrial- and death receptor-mediated pathways, and alterations of mitochondrial cardiolipin are now recognized to be involved in the development of diabetes and several other pathologic conditions [29, 33, 34, 48, 49, 55–61]. We have observed that generation of ROS by mitochondria triggers apoptosis in INS-1 insulinoma cells and in mouse pancreatic islet **β**-cells in a process that involves mitochondrial phospholipid oxidation and cytochrome *c* release [57, 62]. 

## 4. ROS Activate Uncoupling Protein 2 (UCP2) through Initiation of Phospholipid Peroxidation in ***β***-Cells

Glucose-stimulated insulin secretion by residual **β**-cells is impaired in subjects with T2DM [7]. Glucose sensing in **β**-cells requires the coupling of glycolysis to oxidative phosphorylation in mitochondria to produce ATP [28]. The respiratory chain complexes pump protons out of the mitochondrial matrix to generate an electrochemical proton gradient that provides the energy required by ATP synthase to produce ATP from ADP. This glucose-stimulated ATP production at the expense of ADP causes the cytoplasmic ATP/ADP ratio to rise, which induces closure of ATP-sensitive potassium channels (K_ATP_), depolarization of the plasma membrane, opening of voltage-gated calcium channels, influx of Ca^2+^, a rise in [Ca^2+^] in cytosol and other cellular compartments, activation of Ca^2+^-sensitive effector elements including the Ca^2+^/calmodulin-dependent protein kinase II**β** and others, and triggering of insulin exocytosis [63]. That oxidative phosphorylation is essential to glucose-stimulated insulin secretion is reflected by the observations, *inter alia*, that specific inhibition of mitochondrial respiratory chain complexes by various means invariably results in blockade of insulin secretion [64]. Moreover, mitochondrial mutations that cause defects in insulin secretion underlie maternally inherited T2DM [65–67]. 

It appears that pancreatic islet **β**-cell mitochondrial membrane potential can be regulated by uncoupling protein-2 (UCP2), which is a member of the mitochondrial anion carrier protein (MACP) family. UCP2 facilitates proton leak to reduce the mitochondrial membrane potential and thus attenuates ATP synthesis. It has been reported that UCP2 negatively regulates insulin secretion and is a major link between obesity, **β**-cell dysfunction, and T2DM [21, 68]. Obesity and chronic hyperglycemia increase mitochondrial superoxide (O_2_^•−^) production [69], and this causes activation of UCP2 and results in pancreatic islet **β**-cell dysfunction [70–73]. Inhibition of UCP2-mediated proton leak by Genipin has been found acutely to reverse obesity- and high-glucose-induced **β**-cell dysfunction in isolated pancreatic islets *in vitro* and in animals with diet-induced T2DM *in vivo* [74, 75]. Together, these observations suggest that activation of UCP2 by superoxide produced by mitochondria could contribute to the development of **β**-cell dysfunction during the evolution of T2DM.

The mechanism by which superoxide activates UCP2 is nonetheless not well understood at present, although studies with probes targeted to subcellular compartments have provided an outline of some possibly contributory processes. Experiments with targeted antioxidants suggest that superoxide or its products activate UCPs on the matrix side of the mitochondrial inner membrane [71]. A study with a mitochondrion-targeted spin trap derived from *α*-phenyl-N-tert-butylnitrone indicated that superoxide activates UCPs via oxidation of unsaturated side chains of fatty acid substituents in mitochondrial phospholipids, for example, cardiolipin, associated with UCPs [76]. In this model, superoxide generated by mitochondria is dismutated by matrix Mn-SOD to hydrogen peroxide (H_2_O_2_), which reacts with Fe^2+^ by the Fenton reaction to generate hydroxyl radical (•OH). The hydroxyl radical extracts a hydrogen atom (H^•^) from a *bis*-allylic methylene moiety of PUFA substituent of a phospholipid, for example, cardiolipin. The resultant carbon-centered radical reacts with molecular oxygen (O_2_) to form a peroxy radical (HC-O-O^•^), which then initiates a chain reaction of lipid peroxidation that results in generation of a complex mixture of products, including 4-hydroxynonenal (*HNE*) and 4-hydroxyhexenal, which activate UCPs [76, 77]. 

Cardiolipin is a major phospholipid constituent of the mitochondrial inner membrane, and the PUFA linoleate is the major fatty acid substituent of **β**-cell cardiolipin [52]. The electron transport chain complexes that generate superoxide reside in the inner mitochondrial membrane, and superoxide production is rate limiting for generating all ROS. Cardiolipin PUFA substituents are especially susceptible to reaction with ROS because of their bisallylic methylene moieties. Like cardiolipin and the electron transport chain complexes, UCP2 also resides in the inner mitochondrial membrane. Together, these observations suggest a sequence in which high rates of mitochondrial superoxide production are associated with correspondingly high rates of cardiolipin oxidation and that this contributes to superoxide-mediated activation of UCPs, perhaps via the generation of HNE and other lipid peroxidation breakdown products. Thus, we propose that cardiolipin oxidation may directly link ROS generation to UCP2 activation and thereby contribute to acceleration of the proton leak that ultimately results in **β**-cell dysfunction. Indeed, it was recently reported that oxidation of a mitochondria-specific phospholipid tetralinoleoyl cardiolipin (L4CL) leads to the formation of 4-HNE via a novel chemical mechanism that involves cross-chain peroxyl radical addition and decomposition [78]. This proposal points to potentially important target processes for the design of interventions to prevent or retard the development of T2DM and perhaps obesity [77].

## 5. The Role of Group VIA PLA_**2**_ (iPLA_**2**_ ***β***) in Remodeling and Repairing Mitochondrial Membranes

Pancreatic islet cardiolipin is enriched in PUFA (89.5%) substituents, including linoleate (71%) [52], and PUFA side chains are especially vulnerable to oxidation because of their bisallylic methylene moieties. Cardiolipin resides in the inner mitochondrial membrane, which is the locus of ROS generation, and this spatial proximity would also favor cardiolipin oxidation under conditions of accelerated ROS production. This susceptibility would be expected to be enhanced in islets, which express low levels of antioxidant enzymes including superoxide dismutase (SOD), catalase, and glutathione peroxidase (Gpx) compared to other tissues, such as liver, kidney, brain, lung, muscles, pituitary gland, and adrenal gland [36, 79–82]. To counteract the continual oxidation of cardiolipin and the associated impairment of mitochondrial function, it thus seems likely that **β**-cells must have some means of repairing or replacing oxidized cardiolipin molecules in order to maintain mitochondrial function. 

It has been proposed that the consecutive actions of mitochondrial phospholipid glutathione peroxidase (PHGPx or Gpx4) and a phospholipase A_2_ (PLA_2_) are required to eliminate oxidized fatty acids from mitochondrial phospholipids under physiological conditions [83]. Gpx4 is a selenoprotein in the glutathione peroxidase (Gpx) family that protects biomembranes, particularly in mitochondria [84]. The PLA_2_ family comprises a diverse group of enzymes that catalyze hydrolysis of the *sn*-2 fatty acyl bond of phospholipids to generate a free fatty acid and a 2-lysophospholipid [85, 86]. Because the PUFAs in phospholipids tend to be located in the *sn*-2 position, it is not surprising that members of the PLA_2_ family can hydrolyze oxidized *sn*-2 fatty acid substituents [85, 87] and are thought to be involved in the repair of oxidized membrane phospholipids [88–90].

Among PLA_2_ family members, Group VIA PLA_2_ (iPLA_2_ **β**) is attracting increasing interest as a potentially critical participant in mitochondrial cardiolipin homeostasis [57, 62, 91, 92]. In eukaryotes, cardiolipin is synthesized *de novo* from phosphatidylglycerol (PG) and cytidine diphosphate-diacylglycerol (CDP-DAG) by cardiolipin synthase on the inner face of the inner mitochondrial membrane [93]. Nascent cardiolipin does not contain PUFAs in its four acyl chains, and the enrichment of PUFA in cardiolipin is thought to be achieved by a remodeling process [94]. Currently, two potential mechanisms, Tafazzin- (*TAZ*-) and iPLA_2_ **β**/MLCLAT-mediated mechanisms, have been proposed to participate in cardiolipin remodeling [93]. 

**Figure 2:**
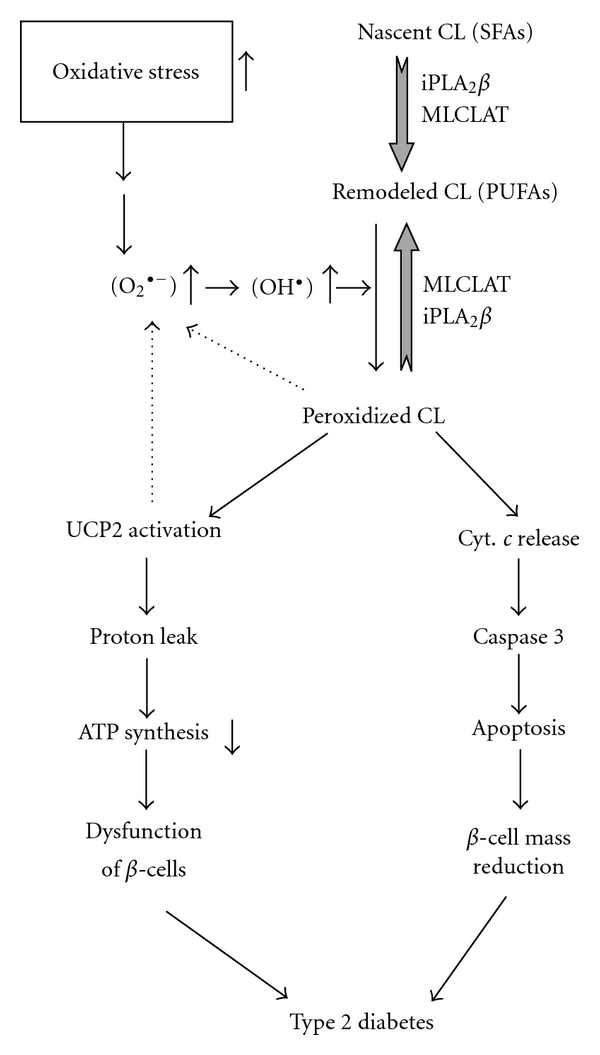
Schematic summary of the proposed role of mitochondrial cardiolipin oxidation in **β**-cell failure in type 2 diabetes mellitus. Oxidative stress results in increased mitochondrial ROS generation in **β**-cells. With moderate oxidative stress, ROS oxidize polyunsaturated fatty acid (PUFA) substituents in mitochondrial cardiolipin molecules, which may generate signals that mitigate ROS production via effects on respiratory electron transport chain complexes or on uncoupling protein 2 (UCP2) (dotted arrows). After delivery of the signal from the ROS-PUFA interaction, the oxidized cardiolipin molecule is repaired in a pathway in which iPLA_2_ **β** excises the oxidized PUFA residue to yielded monolysocardiolipin (MLCL), which is then reacylated with an unoxidized PUFA substituent by MLCL acyltransferase (MLCLAT) to complete the oxidation and repair cycle. Under conditions of overwhelming oxidative stress imposed by high metabolic loads, the rate of cardiolipin oxidation exceeds the capacity of the repair mechanism and oxidized cardiolipin molecules accumulate and compromise mitochondrial membrane integrity, and this leads to cytochrome *c* (Cyt. *c*) release into the cytosol and induction of apoptosis, which eventuates in **β**-cell failure and the development of T2DM. Circumstances in which the capacity of the repair mechanism is overwhelmed in this way would include reductions in iPLA_2_ **β** activity caused by genetic deficiency, pharmacologic inhibition, or yet to be defined regulatory influences on expression. Block arrows denote the iPLA_2_ **β**-mediated deacylation; line arrows denote the stimulatory pathway. SFAs: saturated fatty acids.

In the *TAZ* pathway, newly synthesized cardiolipin is proposed to be deacylated and reacylated by *TAZ*. It appears that this mechanism is essential for optimal mitochondrial function in heart because Barth Syndrome, which is characterized by a severe cardiomyopathy [95, 96], is caused by a mutated *TAZ* gene that encodes a putative mitochondrial phospholipid acyltransferase with both deacylation and reacylation activities [95, 97]. In the iPLA_2_ **β**/MLCLAT-mediated pathway, newly synthesized cardiolipin is proposed to be deacylated by iPLA_2_ **β** to MLCL that is reacylated to cardiolipin by a MLCL acyltransferase (MLCLAT) (Figure 2). It has recently been recognized that mutations in the PLA2G6 gene that encodes iPLA_2_ **β** underlie the neurodegenerative disease infantile neuroaxonal dystrophy (INAD) [98] and that a similar disorder develops in mice with a disrupted *iPLA_2_*β** gene (Malik et al. [99]). It has been suggested that iPLA_2_ **β** also plays a role in cardiolipin remodeling both in a *Drosophila *model of the Barth Syndrome [92] and in the spontaneously hypertensive rat heart failure model [91]. 

 We have also reported observations that are consistent with a role for iPLA_2_ **β** in **β**-cell mitochondrial function that include that iPLA_2_ **β** resides in mitochondria in INS-1 insulinoma cells and that its activity provides protection against the effects of staurosporine to induce loss of mitochondrial membrane potential, release of cytochrome *c* and Smac/DIABLO into cytosol, peroxidation of mitochondrial membranes, and apoptosis [62]. Staurosporine is an inhibitor of various isoforms of Protein Kinase C and strongly stimulates mitochondrial generation of ROS [100].

Both Barth Syndrome and INAD are human genetic disorders that are often fatal in childhood [95, 98] at an age before type I DM might be manifest, which requires loss of about 80–90% of the islet **β**-cell mass at the age of onset [101]. Animal models that have been used to evaluate the potential involvement of iPLA_2_ **β** in disease processes include administration of a suicide substrate bromoenol lactone (BEL) inhibitor of iPLA_2_ **β** [102] and iPLA_2_ **β**-null (iPLA_2_ **β** ^−/−^) mice generated by homologous recombination to disrupt the *iPLA_2_*β** gene [103]. These iPLA_2_ **β**-null mice develop a disorder similar to INAD [99, 104], exhibit several other phenotypic abnormalities [103, 105–113], and have permitted evaluation of the role of iPLA_2_ **β** in **β**-cell failure *in vivo* [57, 103, 114, 115]. 

We have observed that acute pharmacologic inhibition of iPLA_2_ **β** in mice impairs glucose tolerance by suppressing insulin secretion and that insulin sensitivity is not affected under these conditions, which suggests that iPLA_2_ **β** deficiency adversely affects glucose-induced insulin secretion by **β**-cells [102]. Consistent with that interpretation, studies with iPLA_2_ *β* ^−/−^ mice that are genetically deficient in iPLA_2_ **β** expression because of homozygous disruption of the *iPLA_2_*β** gene by homologous recombination [103] have revealed that they exhibit greater impairment in islet function, as reflected by fasting blood glucose levels and glucose tolerance testing responses, than do wild-type mice in response to metabolic stress imposed by low-dose streptozotocin (STZ) treatment, by consumption of a high-fat diet, or by staurosporine administration [57, 114, 115]. 

Moreover, findings with pancreatic islets isolated from iPLA_2_ *β* ^−/−^ mice corroborate the involvement of iPLA_2_ **β** in glucose-stimulated insulin secretion because iPLA_2_ *β* ^−/−^ islets exhibit diminished secretory responses compared to wild-type islets [57, 114, 115]. In addition, incubation with elevated concentrations of glucose and free fatty acids *in vitro* results in higher levels of **β**-cell apoptosis and of peroxidation of mitochondrial membrane phospholipids with islets isolated from iPLA_2_ *β* ^−/−^ mice compared to those from wild-type mice [57]. These findings suggest that iPLA_2_ **β** plays an important role in maintenance of **β**-cell mitochondrial membrane integrity and that iPLA_2_ **β** deficiency increases **β**-cell susceptibility to injury by ROS generated by mitochondria in response to metabolic stress [57, 115]. This could lead to increased vulnerability to induction of apoptosis under conditions of metabolic stress that lead to **β**-cell failure and T2DM [57, 115]. **β**-cell mitochondrial membrane peroxidation is also more readily induced under conditions in which iPLA_2_ **β** is inhibited pharmacologically with the suicide substrate BEL [57].

It has been suggested that oxidation of PUFA in mitochondrial cardiolipin and other phospholipids may serve to trap ROS in order to protect mitochondrial proteins or DNA from oxidative injury or that reaction of PUFA with ROS may generate signals to respiratory chain proteins and UCP2 that mitigate ROS generation and increase proton leak [49, 77, 116–118]. A repair mechanism in which iPLA_2_ **β** excised oxidized fatty acid substituents from mitochondrial cardiolipin and other phospholipids would generate monolysocardiolipin (MLCL) that could be reacylated with an unoxidized PUFA substituent might complete a cycle that could modulate the levels and effects of ROS during stress responses. 

Under conditions in which the rates of ROS generation and oxidation of PUFA in mitochondrial cardiolipin and other phospholipids exceed the capacity of the repair system, accumulation of oxidized phospholipids could eventually impair the integrity of mitochondrial membranes and result in release of cytochrome *c* into cytosol and induction of **β**-cell apoptosis. One circumstance in which the capacity of this repair system would be reduced is when iPLA_2_ **β** activity is low because of pharmacologic inhibition, genetic deficiency, or still to be defined regulatory influences. Under such conditions, accumulation of oxidized mitochondrial phospholipids and leakage of cytochrome *c* could result in accelerated induction of apoptosis that ultimately leads to **β**-cell failure and T2DM (Figure 2).

Of interest in this regard are findings with the *db/db* mouse, which is a model of obesity, dyslipidemia, and diabetes in which there is a defective leptin receptor. Islets isolated from *db/db* mice express lower levels of iPLA_2_ **β** than do islets from control mice [57], and this could impair cardiolipin remodeling and repair in *db/db *β**-cells and increase their susceptibility to oxidative injury, which could accelerate obesity-associated **β**-cell loss and the development of T2DM.

## 6. Conclusions and Therapeutic Implications

Modification of mitochondrial cardiolipin molecular species by oxidation and other processes is now recognized to be associated with many human diseases, including diabetes mellitus [55, 58, 60]. Cardiolipin is a critical structural component of mitochondrial membranes and plays important roles in regulating ATP synthesis and the mitochondrial pathway of apoptosis [49, 119]. Metabolic stresses imposed by obesity and hyperglycemia are often accompanied by increased rates of mitochondrial ROS production [69]. PUFAs are especially susceptible to oxidation by ROS because they contain a highly reactive bisallylic methylene moiety from which hydrogen is readily abstracted to yield a center for initiation of peroxidation chain reactions, and cardiolipin is enriched in PUFA substituents. 

A repair mechanism in which iPLA_2_ **β** excises oxidized PUFA substituents of cardiolipin to yield an MLCL intermediate that can be reacylated with an unoxidized PUFA substituent may be critical for the maintenance of mitochondrial membrane integrity, and it seems likely that some such repair mechanisms would be necessitated by the close spatial proximity of mitochondrial cardiolipin to the locus of ROS generation. Failure of this repair mechanism could compromise mitochondrial membrane integrity and facilitate release of cytochrome *c* into cytosol and induction of apoptosis. Observations from several laboratories [57, 62, 91, 92, 102, 114, 115] suggest that iPLA_2_ **β**-catalyzed deacylation participates in a cardiolipin remodeling and repair cycle that maintains an optimal mitochondrial functional status in **β**-cells. 

Reduced iPLA_2_ **β** activity resulting from genetic deficiency, as in INAD patients or iPLA_2_ **β** ^−/−^ mice, or downregulated expression, as in *db/db* mouse islets, could impair this cardiolipin repair mechanism and result in accumulation of oxidized cardiolipin species that compromise mitochondrial membrane integrity. The ensuing release of cytochrome *c* into cytosol and induction of apoptosis might result in the neurodegeneration in INAD and in **β**-cell loss during the development of T2DM. Further study of cardiolipin remodeling and repair and the role of iPLA_2_ **β** in these processes could increase our understanding of the pathogenesis of diabetes mellitus and neurodegeneration and suggest novel strategies for design of therapeutic interventions to prevent or retard the development of T2DM and neurodegenerative diseases in humans. 

An example of such a potential intervention would be administration of an agent that accumulated in mitochondria and protected them from injurious effects of ROS. The antioxidant NtBHA accumulates in mitochondria, and we have found that it attenuates staurosporine-induced apoptosis and prevents peroxidation of mitochondrial phospholipids in islets from iPLA_2_ *β* ^−/−^ mice [57]. A similar approach to protecting mitochondrial cardiolipin and other phospholipids from oxidation might represent an attractive therapeutic strategy in humans with metabolic or neurodegenerative diseases. Such approaches might be complicated by the fact that some effects of ROS are not injurious but represent essential signaling roles in physiological regulatory mechanisms. For example, mitochondrial ROS generation has been suggested to be an essential signal in the glucose-stimulated insulin secretory pathway in **β**-cells and also to be involved in insulin signaling and sensitivity [120]. 

Thus, manipulating ROS production or interaction with intracellular targets *in vivo* could have unexpected and unwanted adverse effects, and the ability to target such interventions with high selectivity to specific intracellular processes, such as inhibition of mitochondrial phospholipid oxidation, might be desirable. It is of interest in this regard that specific delivery of antioxidants to mitochondria, such as mitoquinone (Mito-Q) and mitovitamin E (mitoVit-E), has been demonstrated to reduce oxidative stress and to improve cardiac function [121, 122] and might be similarly beneficial in **β**-cells. In addition, melatonin specifically inhibits mitochondrial cardiolipin oxidation and has also been found to prevent induction of the mitochondrial permeability transition (MPT) and release of cytochrome into cytosol and to protect against myocardial ischemia-reperfusion injury [120, 123].

##  Disclosure

The authors have nothing to disclose.
